# Factors Influencing the Mental Health of Caregivers of Children with Cerebral Palsy

**DOI:** 10.1192/j.eurpsy.2024.446

**Published:** 2024-08-27

**Authors:** R. Alhumaidah

**Affiliations:** College of Medicine, King Saud bin Abdulaziz University for Health Science, Jeddah, Saudi Arabia

## Abstract

**Introduction:**

Cerebral palsy refers to a heterogeneous group of non-progressive neurodevelopmental disorders manifesting in infancy or childhood and varying in severity. It characterized by impaired motor function, sensation, and depressed intellectual abilities. Functional limitations in patients with cerebral palsy may result in chronic dependency, thereby compromising caregivers’ mental health and interfering with the integrity of the family structure.

**Objectives:**

This study aimed to determine the different factors affecting the mental health of caregivers of children with cerebral palsy and to raise awareness among healthcare providers.

**Methods:**

A cross-sectional study was conducted among caregivers of children with cerebral palsy in National Guard Health Affairs-Jeddah, Saudi Arabia, using the Depression Anxiety Stress Scale-21, a validated questionnaire assessing: depression, anxiety, and stress. This questionnaire was used to assess the mental health of the caregivers. In addition, factors reflecting child’s health condition, such as visual impairment, number of emergency department visits, and number of Pediatric Intensive Care Unit admissions were reported to investigate the impact on the caregiver’s mental health.

**Results:**

The sample included 40 caregivers, of which 72.5% were mothers. According to the Depression Anxiety Stress Scale-21 score, 12.5% (n = 5) of the caregivers had moderate depression scores, 10% (n = 4) revealed extremely severe depression, and 10% (n = 4) showed moderate anxiety. Furthermore, 12.5% (n = 5), 15% (n = 6), and 7.5% (n = 3) of the caregivers have scored as moderate, severe, and extremely severe stress levels, respectively. Caregivers’ depression, anxiety, and stress scores were significantly (p ≤ 0.05) associated with the impact of vision of their dependent children, frequent hospital admissions, and frequent emergency department visits. Increased Pediatric Intensive Care Unit admissions in the past year were also significantly associated with higher caregiver anxiety scores.

**Conclusions:**

To the best of our knowledge, the dimension of caregivers’ stress and anxiety and their association with the children’s dependency level is not well documented in our region. Caregivers of children with cerebral palsy reported having mental health challenges associated with the children’s vision, frequent need for acute medical care, and hospital admissions.

Healthcare workers should provide early and proactive planning of medical and social support for children and their families using a family-centered approach.

**Disclosure of Interest:**

None Declared

